# High-volume cervical lymph node metastasis in papillary thyroid carcinoma: development and external validation of an interpretable prediction model

**DOI:** 10.3389/fonc.2026.1963925

**Published:** 2026-10-06

**Authors:** Dehao Wang, Jinchao Wang, Zhen Wu, Zhaoqing Cui

**Affiliations:** 1 School of Medicine, Shandong First Medical University, Jinan, China; 2 Department of Thyroid, Breast and Hernia Surgery, Liaocheng People’s Hospital Affiliated to Shandong First Medical University, Liaocheng, China

**Keywords:** calibration, cervical lymph-node metastasis, external validation, machine learning, molecular biomarkers, papillary thyroid carcinoma, SHAP

## Abstract

**Background:**

The transportability of models for postoperative high-volume cervical nodal disease in papillary thyroid carcinoma (PTC) and the incremental predictive value of molecular variables remain uncertain.

**Methods:**

We developed models in the public Deyang cohort (521 patients; 90 events) and performed geographic external validation in the independent Liaocheng cohort (193 patients; 73 events). The outcome was >5 pathologically confirmed metastatic cervical lymph nodes. Five algorithms were compared using repeated stratified nested cross-validation with a prespecified 10-variable postoperative feature set. Ridge logistic regression (C = 0.1) was selected and applied without refitting. Paired comparisons of 8-variable clinicopathological and 10-variable clinicopathological–molecular models assessed the incremental value of high-risk co-mutation status and mutation count in both cohorts.

**Results:**

Internal validation yielded an area under the receiver operating characteristic curve (AUROC) of 0.795 (95% CI, 0.745–0.842), average precision (AP) of 0.497 (0.406–0.598), and Brier score of 0.115 (0.098–0.134). External AUROC was 0.752 (0.678–0.821), AP 0.601 (0.498–0.728), and Brier score 0.217 (0.180–0.254). Observed and mean predicted risks were 37.8% and 23.9%, respectively; the observed-to-expected ratio was 1.582 (1.319–1.860) and the weak-calibration slope was 0.831 (0.542–1.191). At the prespecified threshold of 0.158, sensitivity was 0.795 and specificity 0.600. Net benefit was 0.254 for the model and 0.261 for classifying all patients as high risk (difference, −0.008; 95% CI, −0.051 to 0.033). External paired differences after adding molecular variables were +0.002 for AUROC (−0.005 to 0.010), −0.002 for AP (−0.016 to 0.010), −0.001 for Brier score (−0.002 to 0.000), and +0.009 for net benefit (−0.003 to 0.026).

**Conclusion:**

The model retained moderate discrimination across centers but underestimated absolute risk and did not demonstrate a net benefit advantage over classifying all patients as high risk at the prespecified threshold. High-risk co-mutation status and mutation count showed no clear incremental predictive value. Further validation with standardized lymph-node assessment and patient outcomes is required before clinical application.

## 1 Introduction

The global burden of thyroid cancer continues to rise. GLOBOCAN 2024 estimated nearly 960, 000 new cases worldwide (1). Papillary thyroid carcinoma (PTC) is the predominant histologic subtype of differentiated thyroid cancer, and most patients have favorable disease-specific survival. Yet cervical lymph node status has traditionally been reduced to whether metastasis is present. This binary classification overlooks nodal burden reflected by node count and maximum diameter, disease extent indicated by anatomic distribution, and recurrence risk associated with extranodal extension (2, 3). The 2015 American Thyroid Association (ATA) guidelines distinguished pN1 micrometastases involving ≤5 metastatic lymph nodes, each <0.2 cm in maximum diameter, from higher-volume N1 disease (3). The 2025 guideline update retained the stratifying value of lymph node number. It incorporated this measure into a risk framework that also considers tumor size, involved compartments, extranodal extension, and other postoperative factors (2). Previous studies reported a locoregional recurrence risk of approximately 4% among patients with lower nodal burden. This risk rose to approximately 19% when more than five lymph nodes were positive (4). Among patients with similar numbers of involved nodes, extranodal extension may further alter risk stratification (5). These findings support defining >5 metastatic lymph nodes across the central and lateral neck compartments as a patient-level high-volume outcome. Compared with the binary outcome of any metastasis, this definition retains more information relevant to recurrence risk (2–5).

In other malignancies, genomic information has expanded beyond mechanistic characterization into clinical risk stratification. The breast cancer 21-gene recurrence score and prostate cancer genomic classifiers (6) add molecular markers to clinical parameters. For some patients, this information can alter prognostic assessment and treatment decisions (7, 8). PTC also has an established molecular basis for stratification. The Cancer Genome Atlas (TCGA) identified heterogeneity dominated by BRAF-like and RAS-like signaling. It also showed that tumors with similar driver backgrounds can behave differently (9). Subsequent multicenter studies associated BRAF V600E with recurrence risk. Tumors harboring both BRAF V600E and TERT promoter mutations were more aggressive (10, 11). These molecular features have also been used to predict lymph node metastasis. A study of fine-needle aspiration specimens integrated gene expression, single-nucleotide variants, and fusion data. The resulting model discriminated better than a single molecular classifier or clinical imaging variables (12). The public Deyang cohort also found that high-risk co-mutations of BRAF V600E with TERT, PIK3CA, or TP53 were significantly associated with central lymph node metastasis (13). The focus has therefore shifted from identifying molecular biomarkers independently associated with outcomes to determining their incremental predictive value beyond conventional clinical models (14).

Many models have been developed to predict lymph node metastasis in PTC. They include conventional logistic regression nomograms, ultrasound radiomics, deep learning, multi-algorithm comparisons, and SHAP-based interpretation. Systematic reviews, however, report substantial variation in study populations, predictors, and validation methods, with risk of bias common. A meta-analysis of 107 studies and 134 models found that discrimination was generally moderate and logistic regression remained the most frequently used method (15, 16). Approaches to high-volume nodal disease include dual-center clinical models, multimodal models combining ultrasound or contrast-enhanced ultrasound parameters with BRAF V600E, and comparisons of models incorporating molecular abnormalities (17, 18). Recent work has begun to report geographic external validation, calibration drift, the clinical consequences of fixed thresholds, and SHAP-based interpretation (19, 20). However, these studies have mostly examined any central lymph node metastasis or high-volume metastasis limited to the central compartment. Two questions therefore remain: how to extend prediction from the central compartment to total cervical nodal burden, and how much value molecular variables add beyond clinicopathological information.

Using the full public Deyang cohort, we developed a postoperative model to estimate the probability of high-volume cervical nodal disease in patients with PTC. High-volume disease was defined as more than five pathologically confirmed metastatic lymph nodes across the central and lateral neck compartments. After selecting the final model from multiple candidate algorithms, we applied it without refitting to the independent Liaocheng cohort for geographic external validation. The original Deyang study’s high-volume nodal subanalysis was restricted to patients with lymph node metastasis (13). We therefore also evaluated model performance in the node-positive subsets of both cohorts as a sensitivity analysis. Model evaluation included discrimination, calibration, overall prediction error, exploratory decision-curve analysis, SHAP-based interpretation, and the incremental predictive value of tumor molecular variables.

## 2 Materials and methods

### 2.1 Study design, participants

This was a retrospective study of postoperative risk-stratification model development and geographic external validation for a pathologically detected cervical nodal-burden phenotype. The model was developed and internally validated using the public cohort from Deyang People’s Hospital and subsequently externally validated in an independent cohort from Liaocheng People’s Hospital. The model was designed to estimate the probability of pathologically detected high-volume cervical lymph-node involvement in patients with papillary thyroid carcinoma (PTC). The study was reported in accordance with the TRIPOD+AI statement (21).

Development data were obtained from the public Dryad dataset (DOI: 10.5061/dryad.bvq83bkpx) and its corresponding original study (13, 22). The original study included patients with postoperative pathologic confirmation of PTC who underwent next-generation sequencing of paraffin-embedded tissue between June 2022 and January 2025. All 521 patients in the public dataset had ascertainable outcomes and the required predictors and were included in model development. The Liaocheng external-validation cohort included patients identified between April 2025 and July 2026. During the study period, 204 patients were screened; 11 did not meet the eligibility criteria, leaving 193 patients for external validation. The sample size was determined by the number of eligible patients available during the study period. In the original Deyang study, patients underwent thyroid lobectomy with isthmusectomy or total thyroidectomy, together with central and/or lateral neck lymph-node dissection; the extent of lymph-node dissection was determined according to the 2012 ATA guidelines as reported in that study. In the Liaocheng cohort, all procedures were performed by senior thyroid surgeons, and information on central and lateral neck dissection, the total number of lymph nodes dissected, and the number of metastatic lymph nodes was extracted.

### 2.2 Outcome and predictors

The primary outcome was >5 metastatic cervical lymph nodes confirmed by postoperative pathology; 0–5 nodes were classified as a non-event. The public Deyang data had already categorized metastatic lymph node count as 0, 1–5, or >5. We classified >5 nodes as an event and the other two categories as non-events. In Liaocheng, the patient-level total was summed from postoperative pathologic results for each cervical compartment, using the same >5-node cutoff. Pathological diagnoses and histopathological features in both the Deyang and Liaocheng cohorts were independently reviewed and confirmed by two qualified pathologists.

The primary feature set was prespecified and comprised 10 postoperative clinicopathologic and molecular variables. These were age <45 years, male sex, bilateral lesions, histologic subtype, maximum tumor diameter >1 cm, and multifocality. The remaining variables were grade of extrathyroidal extension, lymphovascular invasion (LVI), high-risk co-mutation, and mutation count. The age and tumor-diameter cutoffs followed the existing definitions in the public dataset. High-risk co-mutation was defined as BRAF V600E coexisting with at least one alteration in TERT, PIK3CA, or TP53. Mutation count was categorized as 0, 1, or ≥2 alterations.

Postoperative formalin-fixed, paraffin-embedded (FFPE) tumor tissue was used for molecular testing in the Liaocheng cohort. Real-time quantitative polymerase chain reaction (qPCR) was performed on an ABI 7500 Real-Time PCR System. Testing used a BRAF/TERT/CCDC6-RET mutation detection kit and a thyroid multigene detection kit. The assay covered BRAF V600E and TERT promoter C228T/C250T mutations. It also covered hotspot mutations in NRAS, HRAS, KRAS, RET, TP53, PIK3CA, and AKT1. Fusion variants included CCDC6-RET, NCOA4-RET, TPM3-NTRK1, PAX8-PPARG, and ETV6-NTRK3. In total, 12 genes and 16 loci were covered. Positive, negative, and internal reference controls were included, and two reviewers independently checked the results. The coefficient of variation for Ct values was required to be ≤5%. Prespecified variable definitions and coding are provided in **Table 1**.

**Table 1:** Prespecified predictors, coding.

Predictor	Prespecified definition and coding
Age <45 years	Age <45 years=1; age >=45 years=0
Male sex	Male=1; female=0
Bilateral disease	Bilateral tumor foci=1; otherwise=0
Histologic subtype	1=classical; 2=follicular; 3=other subtype
Maximum tumor diameter >1 cm	Maximum tumor diameter >1 cm=1; <=1 cm=0
Multifocal disease	Two or more tumor foci=1; unifocal=0
Extrathyroidal extension	0 = none; 1 = microscopic ETE; 2 = gross ETE, following the classification used in the original Deyang study
Lymphovascular invasion (LVI)	Explicit lymphovascular invasion=1; otherwise=0
High-risk co-mutation	BRAF V600E plus at least one alteration in TERT, PIK3CA, or TP53 = 1; otherwise=0
Mutation count	0=no definite alteration; 1=single-gene alteration; 2=two or more gene alterations

ETE, extrathyroidal extension; LVI, lymphovascular invasion.

### 2.3 Data harmonization and preprocessing

Variables in both cohorts were harmonized using common definitions. In Liaocheng, total positive lymph node count was obtained by summing the pathologic counts across cervical compartments. The 521 Deyang and 193 Liaocheng patients had no missing values for the primary outcome or 10 predictors.

Binary variables were standardized, while histologic subtype, grade of extrathyroidal extension, and mutation count underwent fixed-level one-hot encoding. All preprocessing parameters were estimated from the training data and applied to the corresponding validation data. No oversampling, undersampling, or class weighting was used to address class imbalance.

### 2.4 Model development and internal validation

The primary analysis compared Ridge Logistic regression, Elastic-net Logistic regression, a linear support vector machine, random forest, and XGBoost within the prespecified 10-variable feature set. These algorithms covered penalized linear models and nonlinear tree-based models. Three reduced feature sets were examined only in exploratory comparisons and did not inform selection of the primary feature set. Candidate hyperparameters and search ranges are provided in the **Supplementary Methods** in **Supplementary Data S1**.

Internal validation used repeated stratified nested cross-validation. The outer loop was stratified five-fold cross-validation repeated 10 times. Within each outer training set, stratified five-fold inner cross-validation selected the algorithm and hyperparameters. Each outer test fold evaluated the complete model-development process. Model selection considered inner-loop AUROC, Brier score, and model parsimony in that order. The tolerance rules are detailed in the **Supplementary Methods** in **Supplementary Data S1**. After internal validation, the final configuration was selected using the same rules and refitted in all 521 Deyang patients.

### 2.5 Threshold selection and independent external validation

The model’s primary output was the predicted probability of high-volume metastasis for each patient. To define an operating point while prioritizing event detection, we prespecified the threshold with the highest specificity among candidates achieving sensitivity ≥0.80. The threshold was determined solely from repeated cross-fitted predictions in the Deyang cohort. Its internal performance was evaluated by reselecting the threshold within each outer training fold and applying it to the corresponding test fold. The final preprocessing rules, model parameters, and classification threshold were fixed before external validation and then applied directly to Liaocheng.

### 2.6 Model performance and incremental molecular analysis

Discrimination was assessed primarily with AUROC, while average precision (AP) was also reported. Overall prediction error was evaluated with the Brier score and scaled Brier score. Calibration was assessed using the observed event rate and mean predicted risk, the observed-to-expected ratio, calibration-in-the-large estimated from an offset model, the jointly estimated weak-calibration intercept and slope, and a flexible calibration curve. At the prespecified threshold, we calculated sensitivity, specificity, positive predictive value, negative predictive value, accuracy, and the confusion matrix. Exploratory decision-curve analysis (23) compared the model with the mathematical strategies of classifying all patients as high risk or all patients as low risk across threshold probabilities from 0.05 to 0.40. Patient-level bootstrap resampling with 2, 000 iterations estimated 95% confidence intervals for AUROC, AP, Brier score, and calibration metrics. For internal validation, 95% confidence intervals for threshold-based proportions were estimated using patient-cluster bootstrap resampling; for external validation, the corresponding intervals were calculated using the Wilson score method.

The incremental predictive value of the molecular variables was evaluated by comparing an 8-variable clinicopathological model with a 10-variable clinicopathological–molecular model. In the Deyang cohort, paired comparisons were based on patient-level mean probabilities from 10 repeated cross-fitted predictions generated using the same outer-fold partitions, with model selection performed separately within the corresponding inner folds. In the Liaocheng cohort, the two prespecified models were compared directly in the same 193 patients. Differences were calculated as the 10-variable model minus the 8-variable model and were assessed for AUROC, AP, Brier score, scaled Brier score, and net benefit at the common threshold; continuous net reclassification improvement and integrated discrimination improvement were included as supplementary measures. Ninety-five percent confidence intervals were estimated using 2, 000 paired patient-level bootstrap resamples. Calibration results for the two models and the paired incremental comparisons were reported separately.

Given the substantial overlap between HighRiskCoMutation and MutationCount, we also compared the original 10-variable ridge model (M10) with a 9-variable model (M9) that omitted only HighRiskCoMutation. M9 retained MutationCount and the eight clinicopathological predictors. The primary sensitivity analysis used the same ridge configuration (C = 0.1) and preprocessing rules as the main model, with paired predictions generated under matched repeated outer splits. Each model was then fitted in the full Deyang cohort and applied directly to the same 193 Liaocheng patients. To maintain comparability of threshold-based metrics, M9 used the threshold established for M10 during development, without threshold reselection or recalibration during external validation. Performance differences were defined as M10−M9, and 95% confidence intervals were estimated using 2, 000 paired patient-level bootstrap samples. Bootstrap intervals were also used for individual classification rates in this sensitivity analysis. As a supporting robustness check, C was reselected within ridge using the same nested cross-validation structure and matched outer splits (**Supplementary Tables S4**–**S8**, **S11**).

Further sensitivity and exploratory analyses were performed as follows. In the Liaocheng cohort, the numbers of lymph nodes dissected and metastatic lymph nodes were summarized, and model discrimination was reassessed among patients with at least six lymph nodes dissected. Alternative outcomes defined as more than three and more than ten metastatic lymph nodes were examined, with event rates, AUROC, and AP reported. To align the analysis with the node-positive population of the original Deyang study, the previously established full-PTC score was evaluated among patients with at least one pathologically confirmed metastatic lymph node, using repeated cross-fitted predictions in Deyang and unadjusted external predictions in Liaocheng. Clinicopathological characteristics were also compared between false-negative and true-positive cases. Recorded compartment-specific variables in Deyang were also used to describe the distribution of central and lateral neck metastases and characterize the composition of the outcome.

Between-cohort comparisons in **Table 2** used Pearson’s chi-squared test, with Fisher’s exact test used for binary variables when any expected cell count was <5. P values were adjusted using the Benjamini–Hochberg procedure across a single family of 11 variable-level comparisons (**Supplementary Table S3**). Seven binary variables (age, sex, bilateral disease, tumor size, multifocality, LVI, and high-risk co-mutation) were compared using 2×2 contingency tables. Four three-category variables (histologic subtype, extrathyroidal extension, mutation count, and nodal-burden category) were each evaluated using an omnibus test of a 2×3 contingency table. The 19 displayed rows in **Table 2** represent 10 predictors and one outcome distribution; individual category levels were not tested separately.

**Table 2:** Comparison of the Deyang development and Liaocheng validation cohorts.

Characteristic/level	Deyang development (n=521)	Liaocheng validation (n=193)	Effect size	Raw P value	BH-adjusted P value
Age <45 years	341 (65.5%)	86 (44.6%)	|SMD|=0.430	<0.001	<0.001
Male sex	144 (27.6%)	36 (18.7%)	|SMD|=0.214	0.014	0.015
Bilateral disease	109 (20.9%)	74 (38.3%)	|SMD|=0.389	<0.001	<0.001
Histologic subtype: classical	498 (95.6%)	100 (51.8%)	V=0.559	<0.001	<0.001
Histologic subtype: follicular	16 (3.1%)	13 (6.7%)	—	—	—
Histologic subtype: other or mixed	7 (1.3%)	80 (41.5%)	—	—	—
Maximum tumor diameter >1 cm	231 (44.3%)	114 (59.1%)	|SMD|=0.298	<0.001	<0.001
Multifocal disease	119 (22.8%)	101 (52.3%)	|SMD|=0.639	<0.001	<0.001
Extrathyroidal extension: none	109 (20.9%)	21 (10.9%)	V=0.172	<0.001	<0.001
Extrathyroidal extension: microscopic	393 (75.4%)	151 (78.2%)	—	—	—
Extrathyroidal extension: gross	19 (3.6%)	21 (10.9%)	—	—	—
Lymphovascular invasion (LVI)	45 (8.6%)	20 (10.4%)	|SMD|=0.059	0.477	0.477
High-risk co-mutation	20 (3.8%)	25 (13.0%)	|SMD|=0.333	<0.001	<0.001
Mutation count: none	29 (5.6%)	7 (3.6%)	V=0.134	0.002	0.002
Mutation count: one	461 (88.5%)	159 (82.4%)	—	—	—
Mutation count: >=2	31 (6.0%)	27 (14.0%)	—	—	—
Pathologically detected nodal burden: no nodal metastasis	206 (39.5%)	14 (7.3%)	V=0.330	<0.001	<0.001
Pathologically detected nodal burden: 1–5 metastatic nodes	225 (43.2%)	106 (54.9%)	—	—	—
Pathologically detected nodal burden: >5 metastatic nodes	90 (17.3%)	73 (37.8%)	—	—	—

Values are n (%). BH, Benjamini–Hochberg; SMD, standardized mean difference. BH adjustment was applied to one family of 11 variable-level P values (seven 2×2 comparisons and four 2×3 omnibus tests), rather than to the 19 displayed rows. For multicategory variables, the omnibus P value is shown only in the first row; “—” in subsequent rows indicates that no separate test was performed. The complete test list is provided in **Supplementary Table S3**.

### 2.7 Model interpretation and statistical software

For the final model fitted in all 521 Deyang patients, SHAP (SHapley additive explanations) LinearExplainer quantified each predictor’s contribution to model output (24). For one-hot encoded categorical variables, components belonging to the same original variable were combined for variable-level interpretation. Global importance was expressed as the mean absolute SHAP value, with 95% confidence intervals estimated by patient-level bootstrap resampling. Signed SHAP values described the direction of each variable’s contribution to prediction. Paired SHAP explanations of M10 and M9 used an Independent masker and the same 150 Deyang patients as the background sample. Mean absolute and mean signed SHAP contributions were compared at the original-variable level (**Supplementary Table S10**; **Supplementary Figure S8**).

Statistical analyses were conducted in Python 3.12, mainly using scikit-learn (25), XGBoost (26), SHAP, statsmodels, and Matplotlib. Software versions, hyperparameter ranges, random seeds, threshold implementation, and SHAP background-sample settings are provided in the **Supplementary Methods** in **Supplementary Data S1**.

## 3 Results

### 3.1 Participants and cohort characteristics

The Deyang development cohort included 521 patients with PTC, of whom 90 had high-volume nodal disease (17.3%). In the Liaocheng cohort, 204 patients were screened and 11 were excluded, leaving 193 patients for external validation; 73 had high-volume nodal disease (37.8%, 95% CI 31.3%–44.8%) and 120 did not (**Figure 1**). The primary outcome and model predictors were complete for all patients, whereas the total number of lymph nodes dissected was missing for one patient. Compared with the Deyang cohort, the Liaocheng cohort had lower proportions of patients aged <45 years (44.6% vs 65.5%; |SMD|=0.430) and of male patients (18.7% vs 27.6%; |SMD|=0.214), but higher proportions with bilateral disease (38.3% vs 20.9%; |SMD|=0.389), a maximum tumor diameter >1 cm (59.1% vs 44.3%; |SMD|=0.298), multifocal disease (52.3% vs 22.8%; |SMD|=0.639), and high-risk co-mutation (13.0% vs 3.8%; |SMD|=0.333). The distribution of histological subtypes also differed between cohorts (Cramér’s V = 0.559) (**Table 2**; **Supplementary Figure S3**).

**Figure 1:**
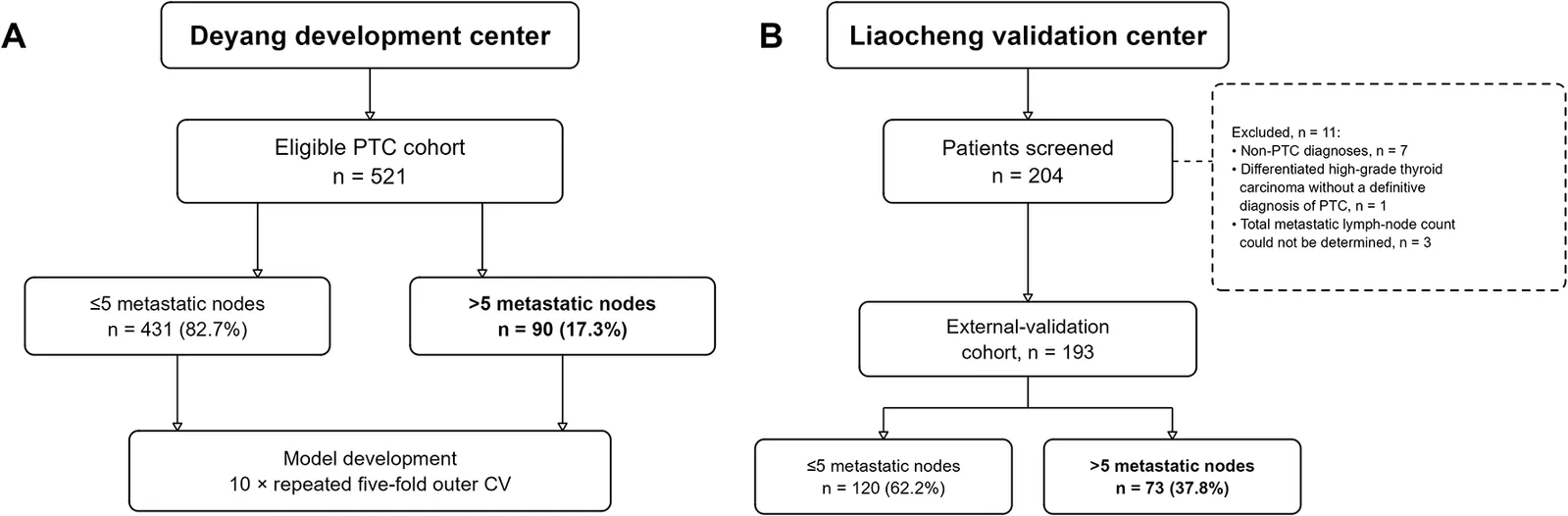
Study design and cohort flow. **(A)** The Deyang development cohort contained 521 patients, including 90 with >5 pathologically detected metastatic cervical lymph nodes. **(B)** Across the two-stage Liaocheng screening process, 204 patients were assessed and 11 were excluded: seven with non-PTC diagnoses, one with differentiated high-grade thyroid carcinoma without a definitive diagnosis of PTC, and three for whom the total number of metastatic lymph nodes could not be determined. The final external-validation cohort included 193 patients, comprising 73 events and 120 non-events. The updated source table contained the 162 patients retained in the historical phase plus 34 newly assessed candidates; three of the latter were excluded and 31 were added to the analysis set. PTC, papillary thyroid carcinoma.

### 3.2 Model development and selection

Within the prespecified 10-variable feature set, cross-fitted performance was similar across five algorithm families. AUROC ranged from 0.752 to 0.795, and Brier scores ranged from 0.113 to 0.128. More complex models showed no consistent advantage (**Supplementary Figure S1**; **Table 3**). Ridge Logistic regression (C = 0.1) was selected as the final model according to the prespecified rules for discrimination, prediction error, and parsimony. Hyperparameter search ranges, model-selection rules, and the final fitted model parameters are provided in **Supplementary Data S1**.

**Table 3:** Repeated nested internal-validation performance of candidate algorithms.

Algorithm	Prespecified search space	AUROC	AP	Brier score	Final lock
Ridge logistic regression	L2; C = 0.1, 1, or 10	0.794	0.501	0.113	Yes; C = 0.1 after separate full-development inner selection
Elastic-net logistic regression	C=0.1, 1, or 10; l1_ratio=0.25 or 0.75	0.795	0.504	0.113	No
Linear SVM	Linear kernel with probability output; C = 0.1, 1, or 10	0.752	0.419	0.128	No
Random forest	300 trees; max_depth=3 or unlimited; min_samples_leaf=2 or 10; max_features=sqrt	0.793	0.508	0.120	No
XGBoost	Six prespecified random combinations from the frozen parameter space	0.785	0.465	0.117	No

AP, average precision; AUROC, area under the receiver-operating-characteristic curve. Candidate-family performance is descriptive cross-fitted performance and was not used to select the final model. A separate repeated inner selection on all 521 development patients locked ridge logistic regression with C = 0.1.

### 3.3 Internal validation

Repeated nested internal validation of the complete model-selection process yielded an AUROC of 0.795 (95% CI 0.745–0.842). AP was 0.497 (95% CI 0.406–0.598), and the Brier score was 0.115 (95% CI 0.098–0.134). The calibration intercept was 0.001 (95% CI −0.371–0.411), and the calibration slope was 0.997 (95% CI 0.793–1.267). No evident systematic shift in predicted probabilities was observed (**Figures 2A–C**; **Table 4**).

**Figure 2:**
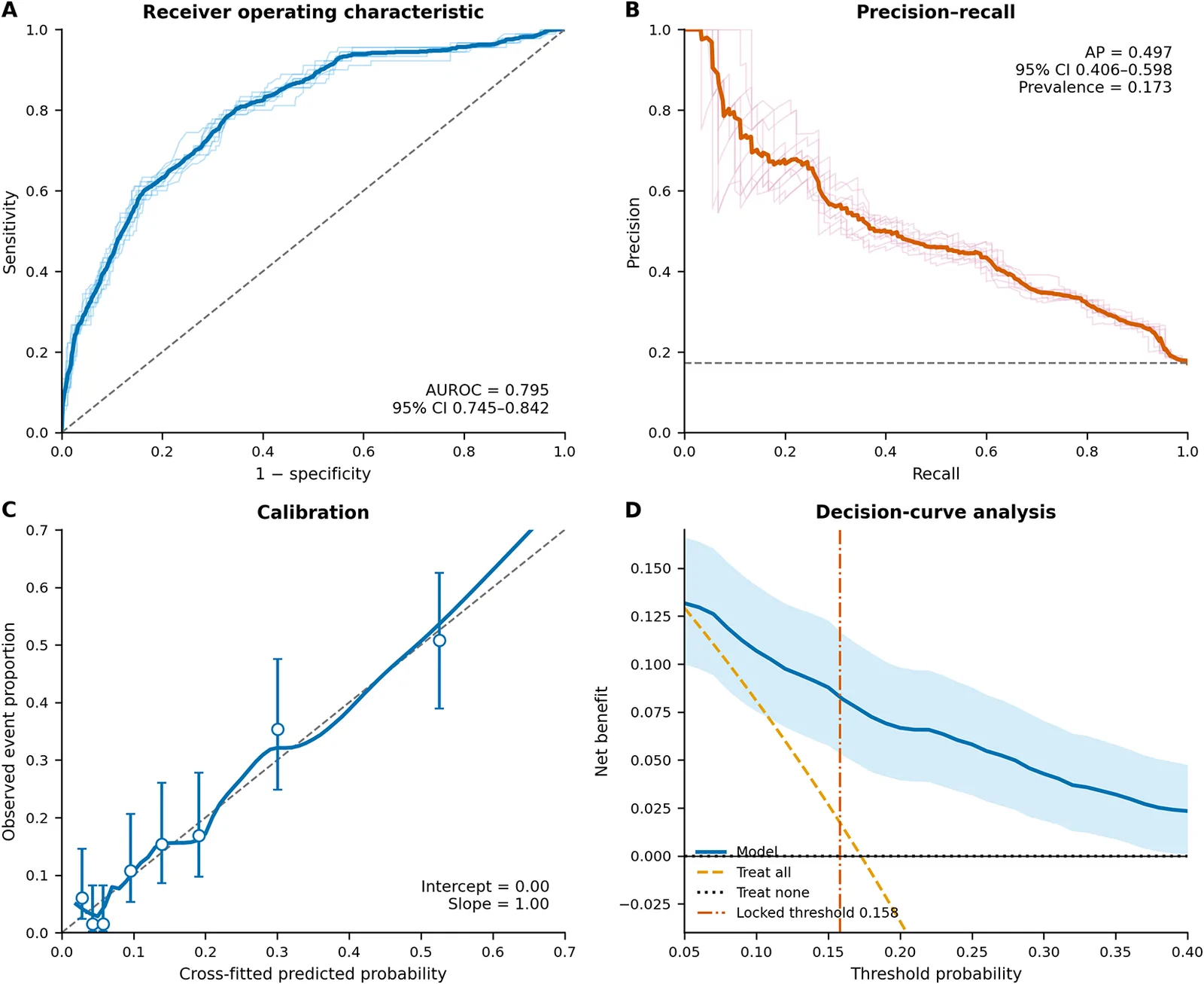
Repeated nested internal validation of the locked modeling strategy in the Deyang cohort. **(A)** Receiver-operating-characteristic curve; AUROC 0.795 (95% CI 0.745–0.842). **(B)** Precision–recall curve; AP 0.497 (95% CI 0.406–0.598), interpreted against an event rate of 0.173. **(C)** Calibration display; joint weak-calibration intercept 0.001 (95% CI −0.371–0.411) and slope 0.997 (95% CI 0.793–1.267). **(D)** Decision-curve analysis over threshold probabilities from 0.05 to 0.40; at the plotted threshold nearest the locked operating point (0.16), model net benefit was 0.082 (95% CI 0.052–0.116) and treat-all net benefit was 0.015 (95% CI −0.021–0.054). AP, average precision; AUROC, area under the receiver-operating-characteristic curve; CI, confidence interval.

**Table 4:** Performance of the locked model in internal and geographic external validation.

Metric	Repeated nested internal validation	Liaocheng external validation
Patients/events	521/90	193/73
Observed event rate (95% CI)	0.173 (0.143–0.208)	0.378 (0.313–0.448)
Mean predicted probability (95% CI)	—	0.239 (0.213–0.266)
AUROC (95% CI)	0.795 (0.745–0.842)	0.752 (0.678–0.821)
AP (95% CI)	0.497 (0.406–0.598)	0.601 (0.498–0.728)
Brier score (95% CI)	0.115 (0.098–0.134)	0.217 (0.180–0.254)
Scaled Brier score (95% CI)	0.197 (0.119–0.272)	0.078 (−0.040–0.191)
Observed-to-expected ratio (95% CI)	—	1.582 (1.319–1.860)
Calibration-in-the-large, offset intercept (95% CI)	—	0.835 (0.487–1.162)
Weak calibration intercept, joint fit (95% CI)	0.001 (−0.371–0.411)	0.629 (0.126–1.178)
Weak calibration slope, joint fit (95% CI)	0.997 (0.793–1.267)	0.831 (0.542–1.191)
Statistical operating point derived from development	Nested threshold procedure	0.158123395
Sensitivity (95% CI)	0.789 (0.704–0.866)	0.795 (0.688–0.871)
Specificity (95% CI)	0.654 (0.614–0.697)	0.600 (0.511–0.683)
Positive predictive value (95% CI)	0.323 (0.264–0.384)	0.547 (0.452–0.639)
Negative predictive value (95% CI)	0.937 (0.910–0.963)	0.828 (0.735–0.893)
Accuracy (95% CI)	0.678 (0.640–0.716)	0.674 (0.605–0.736)
Confusion matrix (TP/FP/TN/FN)	—	58/48/72/15
Model net benefit (95% CI)	0.082 (0.052–0.116) at plotted 0.16	0.254 (0.189–0.320)
Treat-all net benefit (95% CI)	0.015 (−0.021–0.054) at plotted 0.16	0.261 (0.181–0.341)
Net-benefit difference: model minus treat all (95% CI)	—	−0.008 (−0.051–0.033)
Net reduction in high-risk classifications per 100 vs treat all (95% CI)	—	−4.07 (−27.00–17.84)

AP, average precision; CI, confidence interval; DCA, decision-curve analysis; TP/FP/TN/FN, true positive/false positive/true negative/false negative. Internal threshold-based metrics were evaluated using thresholds selected within the corresponding outer training folds; external metrics used the exact locked threshold. The development decision-curve values use the plotted threshold of 0.16, the nearest grid point to the exact locked threshold.

In the nested outer-test threshold procedure, sensitivity was 0.789 (95% CI 0.704–0.866), and specificity was 0.654 (95% CI 0.614–0.697). Positive predictive value was 0.323, and negative predictive value was 0.937. In the development cohort, the decision curve showed a model net benefit of 0.082 (95% CI 0.052–0.116) at 0.16. This was the plotted threshold nearest the locked threshold and exceeded the treat-all net benefit of 0.015 (**Figure 2D**). Other internal performance metrics are reported in **Table 4**.

### 3.4 Independent external validation

In the 193 patients in the Liaocheng cohort, the model achieved an AUROC of 0.752 (95% CI 0.678–0.821), an AP of 0.601 (0.498–0.728), a Brier score of 0.217 (0.180–0.254), and a scaled Brier score of 0.078 (−0.040–0.191). The observed event rate was 0.378 (0.313–0.448), and the mean predicted probability was 0.239 (0.213–0.266). The observed-to-expected ratio was 1.582 (1.319–1.860), the calibration-in-the-large estimated from the offset model was 0.835 (0.487–1.162), and the jointly estimated weak-calibration intercept and slope were 0.629 (0.126–1.178) and 0.831 (0.542–1.191), respectively (**Figures 3A–C**; **Table 4**). At the threshold of 0.158123395, there were 58 true positives, 48 false positives, 72 true negatives, and 15 false negatives. Sensitivity was 0.795 (95% CI 0.688–0.871), specificity was 0.600 (0.511–0.683), positive predictive value was 0.547 (0.452–0.639), negative predictive value was 0.828 (0.735–0.893), and accuracy was 0.674 (0.605–0.736) (**Supplementary Figure S2**; **Table 4**). Compared with the 58 true-positive patients, the 15 false-negative patients were older (median age, 53 vs 40 years), had smaller tumors (median maximum diameter, 1.0 vs 1.9 cm), and were less likely to be aged <45 years (13.3% vs 67.2%) or to have bilateral disease (20.0% vs 60.3%). The net benefit was 0.254 (0.189–0.320) for the model and 0.261 (0.181–0.341) for the classify-all-as-high-risk strategy, corresponding to a difference of −0.008 (−0.051–0.033) (**Figure 3D**; **Table 4**).

**Figure 3:**
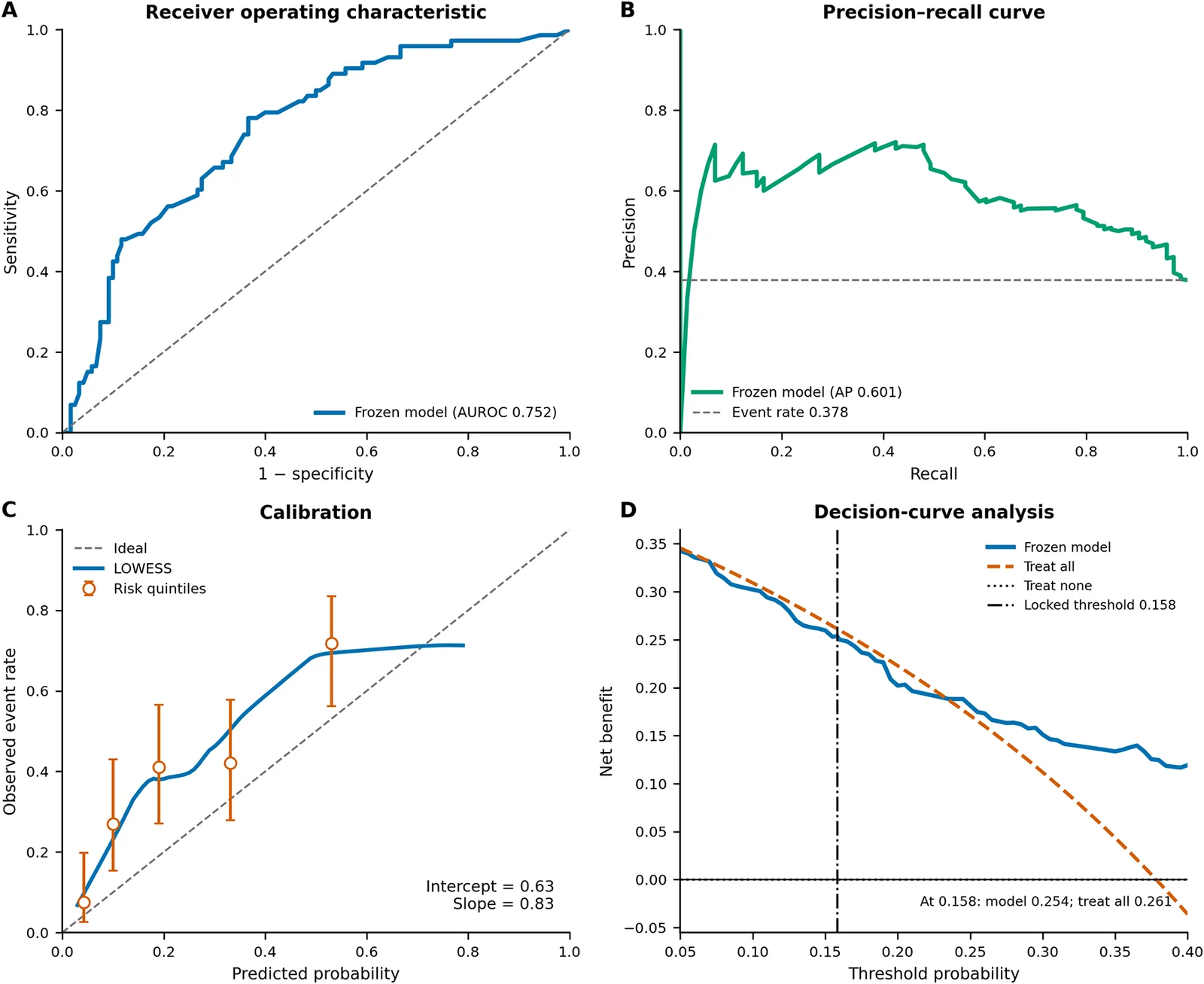
Geographic external validation of the unchanged frozen model in 193 Liaocheng patients. **(A)** AUROC 0.752 (95% CI 0.678–0.821). **(B)** AP 0.601 (95% CI 0.498–0.728), interpreted against an event rate of 0.378. **(C)** Calibration assessment: observed event rate 0.378, mean predicted probability 0.239, observed-to-expected ratio 1.582, offset calibration-in-the-large 0.835, and joint weak-calibration intercept and slope of 0.629 and 0.831, respectively. **(D)** At the locked threshold of 0.158123395, model net benefit was 0.254 and treat-all net benefit was 0.261; the difference was −0.008 (95% CI −0.051–0.033), providing no evidence of a net-benefit advantage over treat-all at this operating point. AP, average precision; AUROC, area under the receiver-operating-characteristic curve; CI, confidence interval.

### 3.5 Lymph-node ascertainment and sensitivity analyses

Among the 193 patients in the Liaocheng cohort, 190 (98.4%) had verifiable records of the extent of lymph-node dissection. All 190 underwent central neck lymph-node dissection, and 73 (38.4%) also underwent lateral neck lymph-node dissection. The mean number of lymph nodes dissected was 15.6 (SD 12.7), with a median of 11.5 (IQR 6–25); this information was missing for one patient. The mean number of metastatic lymph nodes was 5.9 (SD 6.0), with a median of 4 (IQR 2–8) (**Supplementary Table S1**). Among the 148 patients with at least six lymph nodes dissected, 73 had high-volume nodal disease and 75 did not, and the model achieved an AUROC of 0.762 (95% CI 0.680–0.833) (**Supplementary Figure S4**). When the outcome was defined as more than three metastatic lymph nodes, 110 patients met the event definition, with an AUROC of 0.663 (95% CI 0.583–0.738) and an AP of 0.702 (0.610–0.793). When the outcome was defined as more than ten metastatic lymph nodes, 33 patients met the event definition, with an AUROC of 0.708 (0.616–0.791) and an AP of 0.299 (0.201–0.457) (**Supplementary Figure S6**). Among patients with at least one metastatic lymph node, the Deyang cohort included 315 patients, of whom 90 had more than five metastatic nodes and 225 had one to five metastatic nodes. The Liaocheng cohort included 179 patients, of whom 73 had more than five metastatic nodes and 106 had one to five metastatic nodes. Repeated cross-fitted predictions in Deyang yielded an AUROC of 0.735 (95% CI 0.671–0.794) and an AP of 0.562 (0.461–0.664), whereas the corresponding external predictions in Liaocheng yielded an AUROC of 0.740 (0.664–0.809) and an AP of 0.621 (0.515–0.752) (**Supplementary Figure S5**). Based on the recorded compartment-specific information in Deyang, among the 90 patients with more than five metastatic nodes across the whole neck, 19 had central-only metastases, seven had lateral-only metastases, and 64 had metastases in both compartments (**Supplementary Table S9**).

### 3.6 Incremental predictive value of molecular variables

In the Deyang development cohort, high-risk co-mutation was present in 20 of 521 patients. The paired differences between the 10-variable and 8-variable models were +0.00093 for AUROC (95% CI −0.00629 to 0.00845), +0.00003 for AP (−0.01469 to 0.01731), and +0.00094 for the Brier score (−0.00086 to 0.00257). The continuous NRI was −0.030 (−0.238 to 0.185), the IDI was −0.00377 (−0.01010 to 0.00362), and the difference in net benefit at the prespecified threshold was −0.00540 (−0.01271 to 0.00072). In the Liaocheng cohort, high-risk co-mutation was present in 25 of 193 patients. Among the same 193 patients, the paired differences between the 10-variable and 8-variable models were +0.002 for AUROC (95% CI −0.005 to 0.010), −0.002 for AP (−0.016 to 0.010), −0.001 for the Brier score (−0.002 to 0.000), +0.003 for the scaled Brier score (−0.002 to 0.009), and +0.009 for net benefit (−0.003 to 0.026). The continuous NRI was +0.123 (−0.087 to 0.338), and the IDI was +0.001 (−0.001 to 0.004) (**Figure 4**; **Table 5**).

**Figure 4:**
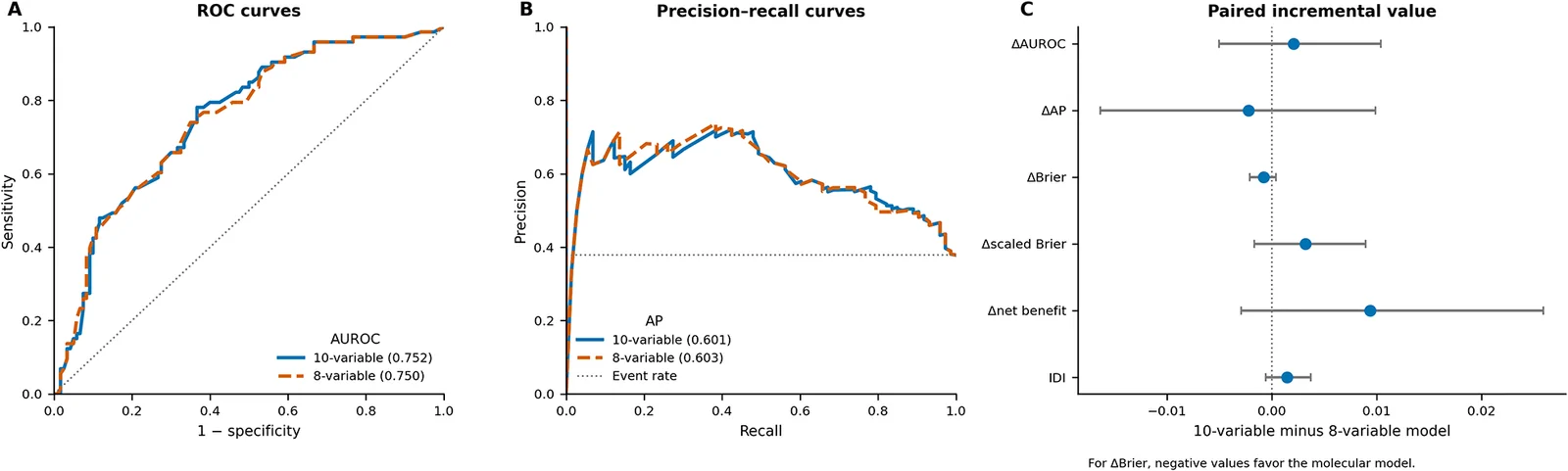
Same-patient external incremental analysis in 193 Liaocheng patients. Predictions from the frozen 10-variable clinicopathologic–molecular model were compared with those from the frozen 8-variable clinicopathologic model without refitting, local tuning, threshold optimization, or recalibration. The panels show **(A)** paired receiver-operating-characteristic curves, **(B)** precision–recall curves, and **(C)** bootstrap differences in AUROC, AP, Brier score, scaled Brier score, net benefit at the common locked threshold, and IDI. Confidence intervals were obtained from 2, 000 paired patient-level bootstrap samples (seed 42). Complete event, non-event, and total continuous NRI estimates are reported together in **Supplementary Table S2** and are not displayed in the main figure. AP, average precision; AUROC, area under the receiver-operating-characteristic curve; IDI, integrated discrimination improvement; NRI, net reclassification improvement.

**Table 5:** Paired incremental value of molecular predictors beyond clinicopathologic variables.

Metric	8-variable clinicopathologic model	10-variable model with molecular predictors	Paired difference (10-variable minus 8-variable; 95% CI)
Panel A. Deyang cross-fitted development comparison			
AUROC	0.793 (0.737–0.842)	0.794 (0.740–0.844)	+0.00093 (−0.00629 to +0.00845)
AP	0.506 (0.406–0.609)	0.506 (0.406–0.611)	+0.00003 (−0.01469 to +0.01731)
Brier score	0.112 (0.094–0.130)	0.113 (0.095–0.131)	+0.00094 (−0.00086 to +0.00257)
Continuous NRI: events	—	—	−0.422 (−0.605 to −0.226)
Continuous NRI: non-events	—	—	+0.392 (+0.304 to +0.478)
Continuous NRI: total	—	—	−0.030 (−0.238 to +0.185)
IDI	—	—	−0.00377 (−0.01010 to +0.00362)
Net benefit at the common locked threshold	—	—	−0.00540 (−0.01271 to +0.00072)
Panel B. Paired Liaocheng geographic external comparison (n=193; 73 events)			
Mean predicted probability	0.239 (0.213–0.266)	0.239 (0.213–0.266)	— (paired difference not formally estimated)
AUROC	0.750 (0.676–0.820)	0.752 (0.678–0.821)	+0.00205 (−0.00503 to +0.01039)
AP	0.603 (0.497–0.729)	0.601 (0.498–0.728)	−0.00220 (−0.01634 to +0.00986)
Brier score	0.218 (0.180–0.255)	0.217 (0.180–0.254)	−0.00076 (−0.00210 to +0.00039)
Scaled Brier score	0.074 (−0.045–0.188)	0.078 (−0.040–0.191)	+0.00322 (−0.00168 to +0.00896)
Net benefit at the common locked threshold	0.244 (0.180–0.309)	0.254 (0.189–0.320)	+0.00939 (−0.00292 to +0.02591)
Observed-to-expected ratio	1.585 (1.323–1.864)	1.582 (1.319–1.860)	— (paired difference not formally estimated)
Calibration-in-the-large (offset intercept)	0.838 (0.486–1.170)	0.835 (0.487–1.162)	— (paired difference not formally estimated)
Weak calibration intercept (joint fit)	0.616 (0.118–1.159)	0.629 (0.126–1.178)	— (paired difference not formally estimated)
Weak calibration slope (joint fit)	0.819 (0.534–1.180)	0.831 (0.542–1.191)	— (paired difference not formally estimated)
Continuous NRI: events	—	—	−0.644 (−0.815 to −0.469)
Continuous NRI: non-events	—	—	+0.767 (+0.643 to +0.875)
Continuous NRI: total	—	—	+0.123 (−0.087 to +0.338)
IDI	—	—	+0.00144 (−0.00059 to +0.00370)

AP, average precision; IDI, integrated discrimination improvement; NRI, net reclassification improvement. Differences are calculated as the 10-variable model minus the 8-variable model. Positive differences favor the molecular model except for Brier score, for which negative differences favor the molecular model. Continuous NRI components and total are reported together.

### 3.7 SHAP interpretation of the final model

SHAP analysis showed that tumor size, lesion distribution, and demographic features primarily drove the final model’s prediction structure (**Figure 5**). Maximum tumor diameter >1 cm had the highest mean absolute SHAP value at 0.664 (95% bootstrap interval 0.656–0.672). Bilateral disease ranked next at 0.348 (0.324–0.372), followed by age <45 years at 0.258 (0.248–0.269). Male sex had a value of 0.230 (0.220–0.240). Together, these four variables accounted for 82.1% of the summed mean absolute contributions across all variables. Mean signed SHAP contributions by feature level indicated that the presence of each of these four features shifted the linear predictor toward high-volume nodal disease. Their corresponding reference levels contributed in the opposite direction.

**Figure 5:**
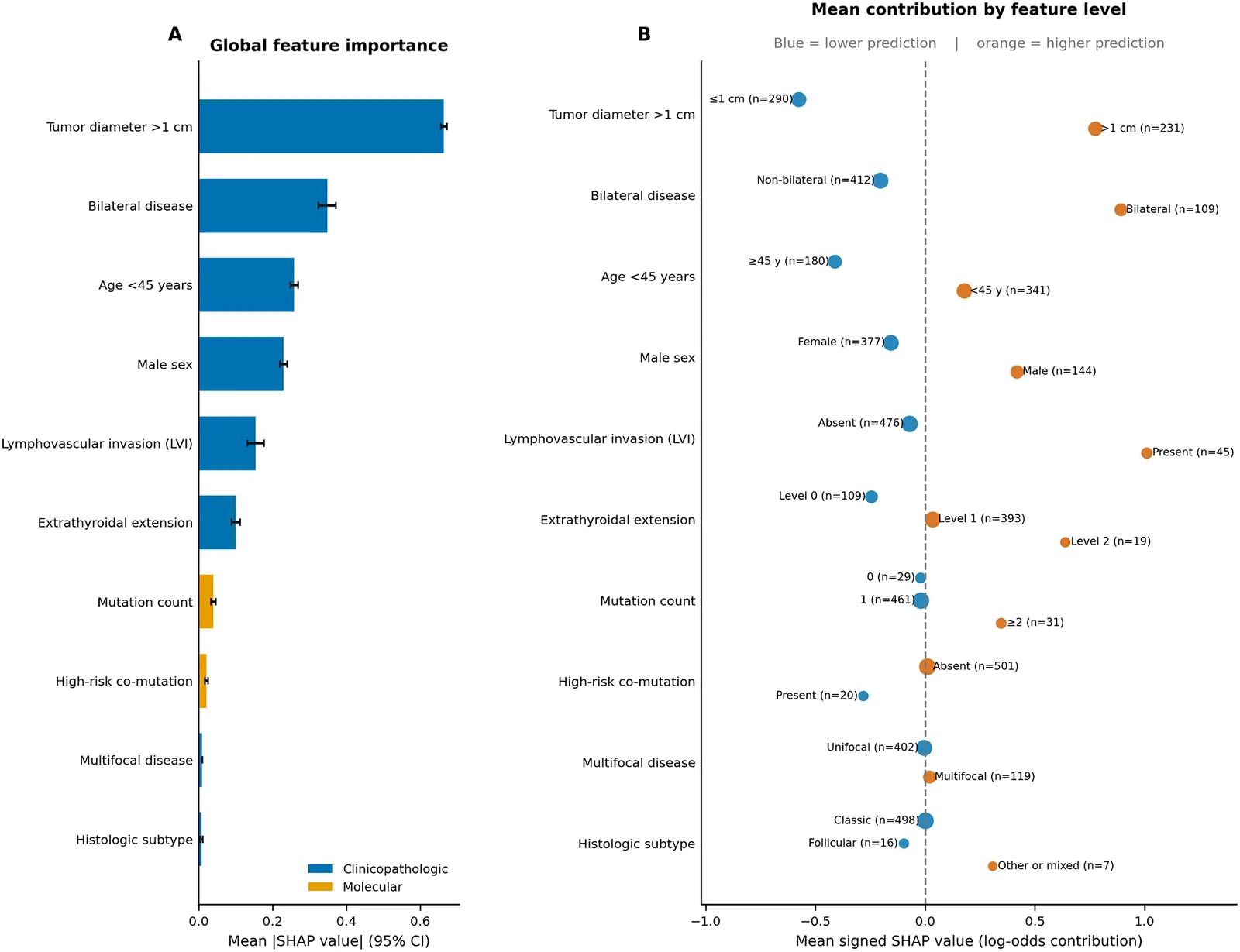
SHAP explanation of the frozen Deyang model. **(A)** Global mean absolute SHAP values and **(B)** feature-level mean signed SHAP contributions describe the contribution of each predictor within the fitted model, including lymphovascular invasion (LVI), to the risk score.

Lymphovascular invasion and gross extrathyroidal extension also produced large positive contributions among affected patients, although their global mean importance was below that of the four leading variables. Molecular variables made smaller overall contributions. Mean absolute SHAP values were 0.039 for mutation count and 0.020 for high-risk co-mutation status. The category of at least two detected molecular alterations contributed positively, whereas the presence of high-risk co-mutations had a negative contribution in the fitted model when included with mutation count. All 20 patients with high-risk co-mutations also had at least two detected molecular alterations. This negative contribution should be interpreted in the context of the substantial overlap between these predictors. Mean contributions from multifocality and histologic subtype were close to zero (**Figure 5**).

### 3.8 Sensitivity analysis excluding high-risk co-mutation

Among patients with MutationCount ≥2, high-risk co-mutations were present in 20 of 31 Deyang patients and 25 of 27 Liaocheng patients, indicating substantial but incomplete overlap between the two variables (**Supplementary Table S4**). After HighRiskCoMutation was omitted, the 9-variable model (M9) achieved an external AUROC of 0.754 (95% CI 0.682–0.822). With paired differences defined as M10−M9, ΔAUROC was −0.0023 (95% CI −0.0083 to 0.0032), ΔAP was +0.0002 (−0.0073 to 0.0095), ΔBrier score was +0.0003 (−0.0017 to 0.0023), and Δnet benefit at the fixed threshold was +0.0136 (−0.0019 to 0.0334). The 95% confidence intervals for all four differences included zero. At this threshold, omitting HighRiskCoMutation increased false negatives from 15 to 18 and reduced false positives from 48 to 46 (**Supplementary Tables S5**, **S11**; **Supplementary Figure S7**).

In the supporting robustness check that reselected C within ridge, both M10 and M9 ultimately selected C = 0.1. Internal-validation results for the fixed-configuration and ridge-only nested analyses are reported in **Supplementary Tables S6** and **Supplementary Table S7**, respectively. In the paired SHAP analysis, the mean signed contribution for MutationCount ≥2 remained positive in M9, and the four leading clinicopathological predictors retained their ranking by mean absolute SHAP value (**Supplementary Table S10**; **Supplementary Figure S8**).

## 4 Discussion

We developed a postoperative risk-stratification model for pathologically detected high-volume cervical nodal disease in the Deyang cohort and performed geographic external validation in 193 patients from Liaocheng. The model retained moderate discrimination in the external cohort but systematically underestimated absolute risk. At the threshold derived from the development cohort, its net benefit did not exceed that of the classify-all-as-high-risk strategy, and 15 of 73 events were classified as low risk. Sensitivity and exploratory analyses addressing lymph-node ascertainment, alternative outcome definitions, and patients with established lymph-node metastasis further characterized model performance under different analytical conditions. Incremental analyses showed that adding high-risk co-mutation status and mutation count to the clinicopathological model did not provide clear incremental predictive value in either the Deyang development cohort or the Liaocheng external cohort. SHAP analysis further showed that model predictions were driven primarily by clinicopathological features, including tumor size, bilateral disease, age, and sex, whereas the two molecular variables made relatively small overall contributions. The sensitivity analysis excluding high-risk co-mutation did not change the overall conclusion. The leading clinicopathological predictors retained their SHAP importance ranking, although the composition of misclassifications at the fixed threshold changed.

Unlike the original Deyang report, we redefined the high-volume outcome. The original study compared 225 patients with 1–5 metastatic nodes and 90 with >5 among 315 patients who had lymph node metastasis. We retained the same 90 events but added 206 patients without metastasis to the reference group. The final analysis compared 431 patients with 0–5 metastatic nodes and 90 with >5 among all 521 patients with PTC (13). This change in the reference group and target population shifted the estimand to the probability of a whole-neck high-volume phenotype among all patients with PTC. Although this estimand differed from that of the original study, tumor size and bilateral disease remained among the highest-ranked features in our SHAP analysis. Their directions were consistent with factors associated with high-volume disease in the original Deyang study (13). Previous high-volume models have also included tumor size, lesion distribution, extrathyroidal extension, and ultrasonographic lymph node features. Most studies, however, focused on cN0 patients or central-compartment outcomes. Differences in target populations and outcome definitions may therefore affect event rates and model discrimination (17–20, 27, 28). Systematic reviews have identified many PTC lymph node prediction models, but validation quality and outcome definitions remain markedly heterogeneous. Logistic regression remains the most common modeling method (15, 16). In multi-algorithm studies, interpretable generalized linear or logistic models have not shown a systematic disadvantage in external cohorts (29, 30). More complex algorithms may therefore offer no greater predictive value in moderately sized samples. The contribution of this study lies in extending prediction to a whole-neck >5-node outcome after redefining the target population and testing the model in a geographically independent cohort.

Genomic studies of PTC have established heterogeneity between BRAF-like and RAS-like molecular lineages. BRAF V600E, TERT promoter mutations, and their coexistence have also been associated with recurrence and other aggressive phenotypes (9–11, 31–33). Models integrating multi-omics information further suggest that molecular data may add value to risk prediction for lymph node metastasis (12). This evidence cannot substitute for paired evaluation of a new marker’s incremental value (14). Our analysis provides direct support for this distinction. We compared an 8-variable clinicopathologic model with a 10-variable model that added high-risk co-mutation status and mutation count under identical outer splits. ΔAUROC was +0.00093 (95% CI, −0.00629 to 0.00845), and ΔAP was +0.00003 (95% CI, −0.01469 to 0.01731). Neither comparison showed a clear improvement in discrimination. ΔBrier was +0.00094 (95% CI, −0.00086 to 0.00257), and prediction error did not decrease. Continuous NRI was −0.030 (95% CI, −0.238 to 0.185). The confidence intervals for IDI and Δnet benefit at the fixed threshold likewise crossed their null values. The original Deyang study associated high-risk co-mutation with the presence of central lymph node metastasis. However, this variable did not distinguish 1–5 from >5 metastatic nodes among patients who already had metastasis (13). Predicting whether metastasis occurs, whether disease becomes high volume, and whether long-term adverse outcomes develop are therefore distinct tasks. SHAP analysis provides model-specific attribution (24). High-risk co-mutation strongly overlapped with a greater burden of molecular alterations. All 20 patients with high-risk co-mutations had ≥2 molecular alterations. HighRiskCoMutation and MutationCount represent a specific high-risk co-mutation pattern and the overall number of molecular alterations, respectively. Their substantial overlap in this sample warrants caution when interpreting the SHAP contribution of either variable alone. The sensitivity analysis excluding HighRiskCoMutation showed no clear additional predictive benefit from retaining it. The leading clinicopathological predictors maintained their SHAP importance ranking, while the composition of misclassifications at the fixed threshold changed. The negative SHAP values therefore need to be interpreted within the fitted model and in the context of these overlapping predictors. Incremental value may be limited by the high prevalence of BRAF V600E in the source cohort and the low frequency of co-mutations. Clinicopathologic phenotypes may also already capture some downstream information.

Cross-center testing further showed that the model retained risk discrimination in a geographically distinct cohort, but calibration drift limited the direct transportability of its absolute-risk estimates. The model showed moderate discrimination in the Liaocheng cohort, with an AUROC of 0.752 (95% CI, 0.678–0.821) and a jointly estimated weak-calibration slope of 0.831 (95% CI, 0.542–1.191). Because the confidence interval crossed 1, there was no clear change in the relative scale of predicted risk. The principal difference concerned baseline risk. The event rate in the Liaocheng cohort was 37.8%, compared with a mean predicted probability of 23.9%, indicating overall underestimation of absolute risk. This was further supported by an observed-to-expected ratio of 1.582 and concordant calibration-in-the-large and weak-calibration intercept estimates. A previous cross-center PTC study also observed marked calibration drift despite preserved external AUROC. Although the direction of drift differed, both findings show that discrimination and calibration can separate after geographic transport (19). The external AP of 0.601 should not be compared directly with the internal AP because this metric depends strongly on baseline event prevalence. External model evaluation should consider discrimination, calibration, and clinical utility rather than rely on AUC alone (34–37). At the prespecified operating threshold of 0.158, sensitivity was 0.795 and specificity was 0.600. Net benefit was 0.254 for the model and 0.261 for the classify-all-as-high-risk strategy, a difference of −0.008 (95% CI, −0.051 to 0.033). Thus, despite retained discrimination, the model did not provide greater net benefit than the classify-all-as-high-risk strategy at this operating point (23, 38). Cross-center transport therefore depends on alignment of target populations and outcome definitions, calibration of absolute risk, and stable decision benefit at prespecified operating thresholds.

The model’s practical use is also constrained by when its inputs become available. Pathologic subtype, extrathyroidal extension, LVI, and tissue molecular results are all obtained postoperatively. NGS in the Deyang cohort likewise used surgical FFPE specimens (13). The model is therefore better considered a postoperative risk-stratification tool for the high-volume phenotype. Its use differs from preoperative decision models based on ultrasonography, fine-needle aspiration cytology, or molecular information from biopsy specimens. A burden of more than five metastatic nodes across the central and lateral neck compartments captures an important dimension of recurrence-risk stratification. Clinical meaning must still be interpreted alongside metastatic focus size, anatomic compartment, extranodal extension, and other pathologic information (2–5). Preoperative applications require models rebuilt around information available before surgery. Postoperative applications should be evaluated in relation to patient outcomes, such as recurrence, and management decisions concerning follow-up strategy and resource allocation (18–20).

The generalizability of this study is limited by its retrospective, two-center data. The public Deyang dataset did not include patient-level counts of examined lymph nodes, precluding direct comparison or adjustment for nodal yield across centers. Although the Liaocheng cohort included 193 patients with 73 high-volume events and provided geographic external validation, uncertainty remained substantial for calibration and threshold-specific net-benefit estimates (39). The high-volume outcome also depends on the extent of lymph node dissection, specimen compartmentalization, and pathologic detection. Between-center measurement differences and case-mix variation may jointly contribute to calibration drift. Their individual effects cannot be separated with the current data. In addition, this study combined positive nodes from the central and lateral neck into a patient-level total-count outcome, although metastases in these compartments differ in their clinical management and prognostic implications. The available data did not support a harmonized compartment-specific external evaluation across the two cohorts, so the findings apply to a postoperative nodal-burden phenotype defined by whole-neck counts. This count-based outcome also does not incorporate the maximum diameter of a nodal metastatic deposit, the number of involved nodal levels, or extranodal extension, and therefore does not fully characterize nodal pathological risk. Molecular information was summarized using high-risk co-mutation status and mutation count. Rare driver events, fusions, variant allele frequencies, and clonal architecture were not modeled. We therefore cannot exclude incremental value from richer molecular features. Although the 10-variable and 8-variable models were compared pairwise in the external cohort, co-mutations were uncommon and the incremental estimates remained imprecise. Thus, the findings support no clear incremental value for these two summary variables, but do not exclude potential value from richer molecular features. Finally, the outcome and major predictors were obtained postoperatively. Direct clinical use still requires validation against explicit downstream decisions and patient outcomes.

Overall, the model retained moderate risk-ranking ability during geographic external validation but systematically underestimated absolute risk, and its net benefit at the prespecified threshold did not exceed that of the classify-all-as-high-risk strategy. High-risk co-mutation status and mutation count did not show clear incremental predictive value beyond clinicopathological information. The model should therefore remain positioned as a postoperative risk-stratification tool and requires further validation in independent cohorts with standardized lymph-node assessment and patient outcomes incorporated into the evaluation.

## Data Availability

The Deyang development dataset is publicly available from Dryad (doi: 10.5061/dryad.bvq83bkpx). The de-identified Liaocheng data are available from the corresponding authors upon reasonable request, subject to applicable ethical, institutional, and data-governance requirements. Supplementary methods and complete fitted model parameters are provided in **Supplementary Data S1**.

## Supplementary material

Supplementary Figure 1 Descriptive comparison of candidate algorithm families across prespecified feature blocks. Cross-fitted AUROC **(A)** and AP **(B)** are shown for Elastic-net Logistic regression, linear support-vector machine, random forest, Ridge Logistic regression, and XGBoost. The vermilion outline identifies the locked expanded-molecular Ridge Logistic configuration (C = 0.1). Final model selection was performed separately using the prespecified repeated inner cross-validation procedure and was not based on selecting the largest value in this heatmap.

Supplementary Figure 2 External risk distribution and locked-threshold classification in the updated Liaocheng cohort. **(A)** Frozen predicted probabilities stratified by the postoperative pathological nodal-burden outcome, with the development-derived threshold of 0.158 shown. **(B)** Confusion matrix at the same threshold: 58 true positives, 48 false positives, 72 true negatives, and 15 false negatives (n=193; 73 events).

Supplementary Figure 3 Differences between the Deyang development and Liaocheng validation cohorts. Absolute standardized mean differences are shown for binary variables and Cramér’s V for multicategory variables; the dashed line at 0.10 is a descriptive reference. Lymphovascular invasion (LVI) is used consistently across cohorts. Colour distinguishes effect-size types and does not denote statistical significance.

Supplementary Figure 4 Sensitivity analyses of the frozen risk score. AUROC estimates with 95% confidence intervals are shown for all eligible Liaocheng patients, the subgroup with at least six examined lymph nodes as a logical minimum-opportunity analysis, and the estimand-bridge population with any pathologically detected nodal metastasis. Restricting the number of examined nodes changes case mix and cannot eliminate nodal-detection bias.

Supplementary Figure 5 LNM-positive estimand bridge using the frozen all-PTC risk score. AUROC and AP are shown among patients with any pathologically detected lymph-node metastasis using repeated cross-fitted Deyang predictions and unchanged external Liaocheng predictions. No conditional model was redeveloped. AP is prevalence dependent and is not directly compared across cohorts.

Supplementary Figure 6 Exploratory alternative outcome cut-offs. AUROC and AP are shown for >3-node and >10-node exploratory outcomes using the frozen >5-node score for ranking only. The primary classification threshold, calibration, and decision-curve interpretations were not transferred to these alternative outcomes.

Supplementary Figure 7 External sensitivity analysis excluding HighRiskCoMutation. The original 10-variable ridge model (M10) was compared with a 9-variable model (M9) omitting only HighRiskCoMutation in the same 193 Liaocheng patients. Both models were fitted in Deyang with C fixed at 0.1 and applied directly to Liaocheng without refitting or recalibration during external validation. **(A)** Receiver-operating-characteristic curves; **(B)** precision–recall curves; **(C)** calibration plots; **(D)** decision curves, with the vertical dotted line indicating the original common threshold of 0.158. Paired performance differences and their 95% confidence intervals are reported in **Supplementary Table S5**.

Supplementary Figure 8 Paired SHAP explanations of M10 and M9. Both models used the same 150 Deyang patients as the background sample and an Independent masker. **(A)** Variable-level mean absolute SHAP values; **(B)** mean signed SHAP contributions for the mutation-count categories. SHAP values are expressed on the log-odds scale. The comparison describes changes in attribution within the fitted models after HighRiskCoMutation was omitted.
