# Supplementary material for: High-volume cervical lymph node metastasis in papillary thyroid carcinoma: development and external validation of an interpretable prediction model
Source: Front Oncol. 2026 Oct 6;16:1963925. doi: 10.3389/fonc.2026.1963925 (PMC13680251; doi:10.3389/fonc.2026.1963925)
Supplement: Supplementary file 1 [file DataSheet1.pdf]

Supplementary Data S1

Supplementary methods and complete fitted model parameters

1. Candidate configurations and selection rules

Ridge logistic regression, elastic-net logistic regression, and the linear support vector machine used C ∈ {0.1, 1, 10}; elastic net additionally used l1_ratio ∈ {0.25, 0.75}. Random forest used 300 trees, max_depth ∈ {3, unlimited}, min_samples_leaf ∈ {2, 10}, and max_features = sqrt.

The XGBoost grid comprised n_estimators ∈ {200, 500}, max_depth ∈ {2, 3}, learning_rate ∈ {0.03, 0.08}, min_child_weight ∈ {3, 8}, reg_alpha ∈ {0, 0.5}, and reg_lambda ∈ {1, 10}, with subsample = colsample_bytree = 0.8. Six configurations were sampled without replacement. The other algorithms used exhaustive grid enumeration, yielding 22 configurations per feature set. Ridge used one fixed seed; the other algorithms averaged predicted probabilities across seeds 17, 2026, and 7919.

Selection retained candidates within 0.01 of the highest mean inner-validation AUROC, then those within 0.005 of the lowest Brier score among these candidates. The preference order was ridge logistic regression, elastic-net logistic regression, linear support vector machine, random forest, and XGBoost, followed by prespecified parameter complexity within each algorithm. Final selection in the full development cohort used five-fold internal validation repeated 10 times.

2. Threshold implementation

With the final configuration fixed, separate five-fold cross-fitting repeated 10 times generated 10 probabilities per patient. These were averaged before applying the threshold rule in main-text Section 2.5. Candidate thresholds comprised unique predicted probabilities plus 0 and 1. Specificity ties were resolved by selecting the highest threshold; probabilities at or above the threshold indicated high risk. Calculations used the full-precision threshold of 0.1581233954272649.

3. SHAP background and interpretation scale

The background comprised 150 Deyang patients sampled without replacement, using an Independent masker. Contributions were expressed on the log-odds scale. Signed one-hot contributions were summed within each patient for each original variable before calculating mean absolute importance. Importance intervals used 2,000 patient-level bootstrap resamples with the model and background fixed.

4. Software versions and random seeds

Analyses used Python 3.12.13, NumPy 2.3.5, pandas 2.2.3, SciPy 1.17.0, scikit-learn 1.8.0, XGBoost 3.4.0, SHAP 0.52.0, statsmodels 0.14.6, Matplotlib 3.10.8, joblib 1.5.3, and openpyxl 3.1.5.

Seeds were: outer splits, 20260806; inner splits, 20260900 + outer split index (0–49); final internal selection, 20260900; parameter sampling and ridge fitting, 20260809; threshold cross-fitting, 20260812; internal primary-performance and threshold bootstraps, 20260810 and 20260814, respectively; internal decision curves, 20260812; Deyang paired incremental bootstrap, 20260813; external validation, 42; and SHAP, 20260811. Seed stability was additionally assessed using 17, 43, 2026, 3407, and 7919.

5. Frozen model parameters and prediction formula

Parameters were exported directly from the frozen models fitted to all 521 Deyang patients. Both models use ridge logistic regression (C = 0.1), with 16 transformed inputs for the 10-variable model and 12 for the 8-variable model. Source-variable definitions and cross-cohort coding follow main-text Table 1.

$\eta = \beta_0 + \Sigma_j \beta_j z_j$; $p = 1 / [1 + \exp(-\eta)]$

For binary variables, $z = (x - \mu)/s$; for categorical variables, $z = I(x = k)$. All category indicators are retained; no additional reference-category term is added. The primary model classifies $p \geq 0.1581233954272649$ as high risk; paired comparisons use this common threshold for both models.

Parameter table A. Frozen intercepts and coefficients.

Transformed term	10-variable model	8-variable model
Intercept / β_0	-1.6106759290237438	-1.7111681355405417
AgeYoung	0.28016969391653085	0.27801566918039489
Male	0.25763570704101701	0.26059386134831813
Bilateral	0.44545508151566532	0.44634099393673271
LargeTumor	0.67099037515206295	0.67034223679529625
Multifocal	0.010088181397968205	0.0086120056082764906
LVI	0.30402659459345066	0.30467845810477517
HighRiskCoMutation	-0.056063313939054962	—
PathologySimple = 1	-0.069673466493552258	-0.068453797388021334
PathologySimple = 2	-0.16792551785220555	-0.17028444271451251
PathologySimple = 3	0.23728230098442632	0.23841285786839581
ETELevel = 0	-0.38734320158621577	-0.38982196398185931
ETELevel = 1	-0.1087786430200319	-0.10592263942920033
ETELevel = 2	0.49580516124491641	0.49541922117692005
MutationCount = 0	-0.12354007285618898	—
MutationCount = 1	-0.12110122385208946	—
MutationCount = 2	0.24432461334694891	—

Parameter table B. Binary-variable standardization parameters.

Variable	Training mean, μ	Scale, s
AgeYoung	0.65451055662188096	0.47552758899184444
Male	0.27639155470249521	0.44721277172240148
Bilateral	0.20921305182341651	0.40674678950195658
LargeTumor	0.44337811900191937	0.49678361747569655
Multifocal	0.22840690978886757	0.4198061378178834
LVI	0.086372360844529747	0.28091311134703578
HighRiskCoMutation	0.038387715930902108	0.19213042236072472

Shared binary predictors use identical standardization parameters in both models. HighRiskCoMutation is included only in the 10-variable model. "—" denotes an excluded term. Categorical levels are PathologySimple = 1/2/3, ETELevel = 0/1/2, and MutationCount = 0/1/2; definitions are in Table 1.

Companion JSON and CSV files retain full-precision machine-readable parameters. Formula predictions were checked against both frozen models in 521 Deyang and 193 Liaocheng patients; maximum absolute probability differences were $\leq 2.23 \times 10^{-16}$. No models were refitted. This verifies parameter export and prediction equivalence, not a complete rerun of model development. No patient-level data are included.
